# Civil Malpractice

**Published:** 1873-04

**Authors:** M. A. McClelland

**Affiliations:** Knoxville, Ill.


A MONTHLY RECORD OF

*Medicine, Surgery and the Collateral Sciences.*

EDITED BY J. ADAMS ALLEN, M.D., LL.D.; AND WALTER HAY, M.D.

---

VOL. XXX. — APRIL, 1873. — NO. 4.

---

### Original Communications.

ARTICLE I.—*Civil Malpractice. A Report presented to the Military Tract Medical Society, at its Fifteenth Semi-Annual Meeting, Jan.* 14, 1873. By M. A. McCLELLAND, M.D., Knoxville, Ill.

(Concluded from page 145.)

The reduction being effected, means must be provided to keep up extension and counter-extension, and also means to prevent lateral and antero-posterior curvature. With the exceptions of fractures of the lower extremities, these ends are accomplished by the use of the splints alone. In fractures of the lower extremities, especially of the femur, we are, except in certain cases, which will be indicated, obliged to use other means for the prevention of shortening. In fractures of the shaft and upper end of the femur, there has been a difference of opinion as to what position the limb should be kept in during the process of repair. Dr. Ashurst in his remarks upon the subject, says: "I have no hesitation in expressing my preference for the treatment of these injuries by means of the *straight position with moderate extension*, whenever that mode of treatment is applicable. In cases of *impacted fracture*, extension is undesirable, and such cases may be treated by position alone, the joint being fixed by means of the long splint, in any of its varieties, or simply supported by means of heavy sand bags placed on either side of the injured member. If the fracture be *unimpacted*, the same treatment should be employed, with the addition of moderate extension. *Counter-extension* may be made by means of a perineal band fastened to the head of the bed, or, which is usually sufficient, simply by elevating the foot of the bed, thus utilizing the weight of the body itself as the counter-extending force. It is right to say that there are certain cases, especially of intra-capsular fracture, in old persons, in which *no apparatus* can be borne, and in which even confinement to bed is fraught with dangerous consequences; under such circumstances the injured limb should be simply laid across pillows as recommended by Sir Astley Cooper, until the pain and inflammation which attend the injury have subsided, the patient being then allowed to get up in a chair or on crutches; bony union, under such circumstances, cannot be hoped for, and the *general* rather than the *local* condition of the patient should be the object of attention." *Princip. and Prac. Surg.*, 259–60.

For the dangers attending an excess of the extending and counter-extending force, the reader will profit by examining the Transactions of the last (May, 1872,) meeting of the American Medical Association, Surgical section. I abridge :—" Dr. Sayre, of New York, thought that the treatment by extension and counter-extension was being carried too far. Every ounce of force applied beyond that necessary to bring the fragments into exact adjustment was injurious; every ounce short of this was insufficient. Dr. Mussey, of Cincinnati, had a case under extension six weeks, with no union. He then took off extension, and union soon followed. A delegate from South Carolina, thought non-union scarcely ever due to over-extension. Non-union was as frequent under the long splint as under the weight and pulley—perhaps more so. Dr. E. M. Moore, of Rochester, thought the weight and pulley gave better results than the straight splint of Desault, but we had become so anxious to avoid all shortening that we often put on too much weight. In transverse fractures, if the limb was kept at its full length, the fragments, though at first in contact, would soon become slightly separated by the absorption of the spicula of bone projecting from their ends; and non-union would ensue. Dr. A. C. Post, of New York, thought the limb might be lengthened by too much extension, even by extension not sufficient to cause discomfort. Examining and measuring all the compound fractures of the thigh under treatment in the Washington Hospitals, toward the close of the war, (where he found Buck's extension giving incomparably the best results), he had discovered one case of slight elongation in an adult. Dr. Gurdon Buck, of New York, claimed for this method, (extension by adhesive plaster, pulley and weight, counter-extension by raising the foot of the bed), which bore his name, that it offered the most efficient means of maintaining uninterrupted extension without discomfort to the patient. It was especially adapted to children. In all his experience but one case of non-union was fairly attributable to over-extension; this was the case of a healthy adult, and the surgeon having the case in charge had, during the whole treatment (ten weeks), kept on a weight of more than twenty pounds. The case had suggested a rule which he had since followed, not to maintain a heavy weight, with hope of securing an unshortened limb, beyond the first fortnight. Dr. Gregory, of Mo., thought that elongation of the limb in young subjects, where the fracture was in the lower third of the femur, might be readily explained by increased growth of the epiphysis from fluxion to the part. Non-union might be due to many other causes, some of them obscure. The obstinacy of Dr. Buck's case would seem to imply that there was some other influence at work to prevent repair than the twenty pounds extension." *Medical Record, June* 15, 1872.

"It is only in fractures of processes, that lengthening of fractured bones would be complained of. The muscular contraction would tend to draw the process away from the shaft or body of the bone, hence the necessity of so dressing the part that the fragments would be kept in as near apposition as possible. For this reason, in fractures of the olecranon, the parts should be kept in the straight position, else by the action of the triceps muscle, the process would be carried so far from the ulna, that the power of extension would be thereafter lost. The great danger to be apprehended in fractures of this joint is anchylosis, and this will occur irrespective of the position in which the arm is dressed." *Hamilton*, 310.

"The tendon of the biceps and facia brachialis in front, and tendon of triceps behind, are all more or less involved in the inflammatory action, set up by fractures in this region, becoming thickened and tense, and more or less adherent to each other and adjoining tissues. Stiffness of the joint is a necessary sequence." *Smith's Op. Surg., vol.* 1, *p.* 761.

To a man with a broken thigh or leg, a comfortable bed to lie upon is of the first importance. "The practitioner who fails to give the proper instruction respecting it, is guilty of gross dereliction of duty." *Gross' Surgery, vol.* 1, *p.* 865. On the same subject, Hamilton remarks, when speaking of fractures of the thigh, (*Frac. and Dis.*, 430): "Where some form of fracture bed cannot be procured, and the patient is compelled to lie on a common cot bedstead, or a common post bedstead, or upon the floor, I cannot think the surgeon ought to be held in any degree responsible for the result." H. H. Smith, (*Op. Surg., vol.* 1, *p.* 606,) is equally decided in respect to this matter. He says: "If the patient has a fractured thigh, leg, or cranium, or any injury by which he is likely to be confined to his bed for some days or weeks, the bed upon which he is to lie, must be prepared for that purpose."

After the reduction of the fracture and the application of those means for the retention of the broken bones in apposition, attention must be given every day or twice a day to the accidents that may follow the injury or the dressing. Paralysis of the bladder, especially in elderly patients, demands attention. Erysipelas, delirium tremens, and tetanus, may also claim his care. The most frequent accidents, however, are bedsores and ulcerations from undue or too long continued pressure. Ulceration is particularly apt to occur upon the heel or upon the internal or external malleolus. This trouble is to be obviated by shifting the pressure to some other part of the limb. The ulceration upon the heel can generally be entirely obviated by a bladder, distended with air or water, placed beneath the heel, and changed as often as necessary, or by a wide rubber band passing beneath the limb and attached to the upper edge of the splints, or even by a piece of cloth attached in the same manner.

Complications. "Implication of the joint in the line of fracture will very often give rise to a certain amount of stiffness if not absolute anchylosis after recovery, or in a strumous constitution may cause disorganization of the articulation, and thus eventually render amputation imperative. Chorea, affecting a limb which is the seat of a fracture, is a very serious complication. Fractures in a paralyzed limb unite; danger to be apprehended here, is from sloughing. Tardy or delayed union of bones is occasionally met with, and is, probably, more often dependent on constitutional than on local causes. Sometimes it appears to result from mere debility and depression without the existence of any positive cachexia. Occasionally, a broken bone does not unite at all, or unites only through the medium of fibrous or ligamentous bands, or, having united, becomes again separated by the absorption and softening of the callus. In some bones, indeed, as in the patella, bony union almost never occurs. So also fractures of the neck of the femur, within the capsule. Among the causes of non-union may be mentioned, general impairment of health, and various cachectic conditions and diatheses, such as scurvy, phthisis, rickets, syphilis, or cancer." *Ashurst, Princip. and Prac. Surg.*

In respect to union in the case of paralyzed limbs, I think, when it takes place, it certainly must be somewhat slower than usual. It is an accepted doctrine, that the nutrition in a paralyzed part is generally if not always impaired, and as a consequence repair must progress slowly. Nutrition might be so much impaired that union would not take place at all. Mr. Travers reports a case in which a "patient had a fracture in the arm and another in the leg, complicated with an injury of the spine which palsied the lower half of the body. The broken humerus readily united, but the tibia and fibula refused to heal." *Gross' Surg., vol.* 1, 3*rd ed., p.* 882.

In suits for malpractice in the treatment of fractures of the lower extremity, one of the allegations is, generally, "shortening." "With regard to the prognosis of fractures through the shaft of the femur, I have no hesitation in saying that I have never seen a *perfect cure*, either in my own practice or in that of others; by this I mean, that I have never seen a cure without shortening. * * * * * * * I have never seen less shortening than a quarter of an inch after fracture of the thigh even in children; and I consider a shortening of from half an inch to an inch, a satisfactory result in adults." *Ashurst's Surg.*, 260.

Velpeau says, that "after fractures of the femur there is no limping unless the shortening exceeds three-quarters of an inch; and the same is true if the shortening occurs in the tibia."

Hamilton, says, (*Princip. and Prac. of Surg.*, 292, 308): "When, in consequence of displacement, an overlapping continues, the average amount of shortening, in adults, in simple fractures, will be about three-quarters of an inch, and ranging from one-quarter of an inch to one inch and a half; nor will a greater amount of shortening necessarily imply unskillful management. With children, the average amount of shortening is probably from one-quarter to half an inch. Compound fractures, including nearly all gunshot fractures, unite generally with a shortening of from one and a half to three inches or more. In fractures of the tibia and fibula, which are mostly oblique, union generally takes place with a shortening of half an inch."

Among eminent surgeons who claim to have cured fractures of the femur without shortening, may be mentioned Amesbury, South, Hunt, and Gamgee, of England; Dorsey, of Philadelphia; and Scott, of Montreal. In regard to these, Prof. Hamilton holds the following language: "It is never a pleasant duty to call in question the accuracy of another's statements as to what he has himself alone seen and done. The circumstances that would justify such an expression of scepticism, where the witnesses, as in this case, are presumed to be intelligent and honest men, must be extraordinary. Such, however, I conceive to be the circumstances in this instance. It is certainly very extraordinary that a few gentlemen of acknowledged skill, but whose means and appliances are concealed from no one, are able to do what nearly the whole world besides, with the same means, acknowledges itself unable to accomplish. Such is the fact, nevertheless; and our lack of faith in their testimony is only a necessary result of our experience, and of the experience of the vast majority of practical surgeons, as opposed to theirs."

In the same connection, Prof. Hamilton gives the names of the many eminent surgeons who admit shortening as a necessary sequence of fractures of the femur. Among these names we find ippocrates, Celsus, Avicenna, Scultetus, Chelius, John Bell, Benj. Bell, Nelaton, Malgaigne, Maclise, Holthouse, Mott, Knight, Detmold, J. Mason Warren, and Lente. Certainly the weight of authority is conclusive. The writer's experience gives seven cases, three of the thigh and four of the leg, in all of which there was shortening.

"It should be borne in mind, that fractured limbs have been released from splints, and other dressings, at the proper time, of proper length and free from deformity, which, nevertheless, have soon become both shortened and bent." *Liston's Elements of Surg.*, *Am. ed.*, 555; also, *Miller's Princip. of Surg.*, 497.

The requisite length of confinement is regulated by the age of the patient, extent of injury, and use to which the part is to be afterward put. In fractures of the lower extremity the time will vary from four to eight weeks, and the limb should not be used for several weeks more.

The following rules for the government of the surgeon in treating fractures, by Dr. Prince, of Jacksonville, are worthy of especial consideration:

"1. Apprise the patient and friends of the difficulties of the case and the uncertainty of a perfect result, whatever skill and attention may be exercised.

"2. Instruct the patient and nurses in the working of the machinery, and direct them to give him immediate notice of anything giving way or seeming to go wrong.

"3. Caution the patient to be careful of the limb after the chief support is taken off, and let him apply a starched bandage or other support to the limb, to be worn long after the patient goes about.

"4. Let him be *sure* that he has *disinterested witnesses* to what he may say and do, so that if any accident or unfavorable result occurs, he may be able to prove his points." *Chicago Med. Journal*, *May*, 1860.

In respect to deformity, it cannot, frequently, be avoided. Especially is this so in dislocations, when one or more of the articular eminences are fractured at the same time. Here, the resulting swelling forbids the discovery of the complication, and indeed should it be discovered, any known mode of dressing would but illy keep the fractured bones in apposition. In dislocations of the clavicle, either of its acromial or sternal ends, deformity is a necessary sequence. So also, fractures of this bone never unite without some deformity. In dislocations of the humerus, the head of the bone may be displaced after perfect reduction, by gradual muscular contraction.

AMPUTATIONS.

In respect to amputations there is much error prevalent. The people suppose that whoever has performed an operation of this character, must necessarily be an eminent surgeon. The profession, on the other hand, is of opinion that almost any one can perform the operation. It is here that errors in judgment should be followed by punishment, if ever. It is not the mechanical skill that would be called in question, as much as the decision in respect to the necessity for the operation. To determine questions on this point requires the highest degree of surgical skill and judgment.

The necessity being answered in the affirmative, we have then to determine at what time the operation should be performed. During "shock," or the period of reaction? Consideration of this question is of the greatest moment to the patient. "Postpone a resort to the knife until there is satisfactory evidence of reaction; until, in a word, warmth and color return to the surface, the pulse beats vigorously at the wrist, and the sufferer regains, in some degree, his consciousness and courage. On the other hand, care is taken not to wait until the part or system are assailed by inflammation, which, under such circumstances, often extends with frightful rapidity, placing the case, perhaps literally, beyond the resources of surgery in the course of a few hours. There is, therefore, a time when interference must be avoided, not less than when it must be courted. The limits of these periods are not always well defined, and hence must be left, in each individual case, to the judgment of the attendant." *Gross' Surg., vol.* I, *p.* 498.

"I am now prepared to affirm, that the period of reaction, or the primary period, is the best point of time for amputation, and that the immediate or period of shock, is not the best, but that, on the contrary, it is an imminently dangerous period; and yet I would make an amputation then, if the patient were bleeding to death, and I could not tie the arteries, or if there were spicula of bone projecting into the nerves and producing spasms; or, if the limb were nearly severed by a cannon ball." *Hamilton, Lecture on Amputations; Med. Record, vol.* 1, *p.* 330. See also, *Pennsylvania Hospital Reports, vol.* 1, *p.* 149.

It will be seen from the foregoing, that "skill" will not be shown, alone, in the mere operation of removing the limb, but underlying and preceding the operation there are questions to be determined of vastly more importance. Nor are the questions just reviewed the only ones. Of no less importance is the question, At what point shall the operation be performed? The older surgeons had points, which they termed "points of election." "As these points have been so frequently changed, indeed, never fully established, modern surgery has formulated a rule upon which to declare the 'place of election.' It is this: inasmuch as the fatality following amputations diminishes as we get farther and farther away from the body, the practice now is very generally adopted, to amputate at that point at which we can save the most of the limb." This rule is *not*, however, applicable to amputations a few inches below the knee; here the "point of election" is the knee joint.

Regard is also had to the method, whether by the circular or flap operation. This is not of so much importance, good results depending more upon the subsequent treatment. As to this, the same general rules applicable to fractures and dislocations are appropriate. The resulting hemorrhage will need the surgeon's especial care.

How frequently should visits be made the patient? Each case will be a law unto itself. The responsibility rests upon the surgeon, and he should make just so many as his judgment dictates, uninfluenced by any inuendoes by the patient or his friends, or else demand that he be released from a responsibility he should not have been asked to assume.

What conclusions may be deduced from these judicial and medical views of "skill" and "negligence"?

Although no surgeon, however eminent, is exempt from the danger of suits from ungrateful patients, the law is for the surgeon's protection as well as for the patient's. No gentleman of this Society need dread to have his professional acts investigated, if he can show that he has acted in good faith, conducting his treatment to the best of his ability; thus far the law is with him. We need nothing more to make our protection perfect, than the enactment of some statute, like that proposed for New York, which will require the plaintiff to secure us against all loss, in case he does not sustain his suit. We should also be permitted to have one expert on the jury, and, too, there should be some way in which the professional standing of the prosecuting witness could be investigated, to the end that such nonsense as that given in the case of *Haire* v. *Reese*—wherein the plaintiff's witness gave it as his opinion, "that the head of the femur might be '*crepitated*' by absorption,"—may not continue to go to juries as sound medical evidence.

Under no circumstances should we compromise such suits. We owe it to ourselves, we owe it to our profession, we owe it to the public whom we serve, to let the matter be tried by the strict letter of the law, and thus vindicate the honor of our profession.

BIBLIOGRAPHY.

Wharton and Stille's Medical Jurisprudence, 2 vols., 3rd ed. Vol. 1—A Treatise on Mental Unsoundness, embracing a general view of Psychological Law. Vol. 2—embracing the topics of Sex, Poisons, Wounds, Identity, Malpractice, etc.

Beck's Medical Jurisprudence, 2 large vols.; embracing all the above subjects except Malpractice. Especially full on Malingering.

Ray's Medical Jurisprudence of Insanity. No better authority on the subject published.

Elwell's Malpractice is the only work yet published upon this specialty. He also discusses the subjects of Insanity and Medical Evidence.

Prof. Stephen Smith, of New York, has been collecting data for a treatise on Malpractice. This, when published, will undoubtedly be the best on the subject.

Ordronaux's Jurisprudence of Medicine in its relations to the Law of Contracts, Torts, and Evidence, is to be consulted by those wishing to know what rights they have in the matters treated of. It contains the statutory enactments relating to the practice of medicine in the States of Alabama, Arkansas, California, Connecticut, Delaware, Florida, Georgia, Kentucky, Louisiana, Maine, Maryland, Massachusetts, Michigan, Minnesota, Mississippi, New Jersey, New York, Ohio, South Carolina, Texas, Vermont, Virginia, Wisconsin, and District of Columbia. Also, Malpractice and the Jurisprudence of Pharmacy, including the Legal Rights of Physician, Druggist and Patient, in Prescriptions.

Dean's Medical Jurisprudence, considers t h relations of—1, Sex; 2, Injuries affecting the organization; 3, Questions arising out of disease; 4, Deceptive Practices, feigning disease; 5, Age, Identity, Life Assurance, and Medical Evidence.

Of Foreign Treatises, Dr. Taylor's Medical Jurisprudence, edited by Penrose, is good authority. He discusses Medical Evidence, Personal Injuries of a Criminal Character, Pregnancy, Infanticide, Legitimacy, Insanity, and Life Insurance.

Dr. Guy's Forensic Medicine, considers questions in respect to Personal Identity, Age, Sex, Impotency, Rape, Pregnancy, Delivery, Fœticide, Infanticide, Legitimacy, Life Assurance, Feigned Diseases, Insanity, Medico-Legal Inspection of the Dead, and Toxicology.

Caspar's Forensic Medicine, in 4 vols., translated and published by the New Sydenham Society, is to be consulted on any of the subjects treated of by the above mentioned authors.

